# Receptor, Signal, Nucleus, Action: Signals That Pass through Akt on the Road to Head and Neck Cancer Cell Migration

**DOI:** 10.3390/cancers14112606

**Published:** 2022-05-25

**Authors:** Albashir Alzawi, Anem Iftikhar, Basher Shalgm, Sarah Jones, Ian Ellis, Mohammad Islam

**Affiliations:** Unit of Cell & Molecular Biology, School of Dentistry, University of Dundee, Dundee DD1 4HN, UK; 160000199@dundee.ac.uk (A.A.); a.iftikhar@dundee.ac.uk (A.I.); b.shalgm@dundee.ac.uk (B.S.); s.j.jones@dundee.ac.uk (S.J.); i.r.ellis@dundee.ac.uk (I.E.)

**Keywords:** tumour microenvironment, extracellular matrix, growth factors, cell migration, Akt, head and neck cancer

## Abstract

**Simple Summary:**

The ecosystem that surrounds a tumour, the microenvironment, has a huge impact on the spread of cancer, but its exact role in the molecular mechanism of spreading is still under scrutiny. This literature review aims to focus on the evidence published on the production of growth factors or proteins from the tumour microenvironment, which initiate signals in cancer cells. This review provides evidence that when Akt, a signalling protein, is activated by different growth factors such as epidermal growth factor, transforming growth factor α/β, vascular endothelial growth factor and nerve growth factor, head and neck cancer cell spreading is stimulated. In a nutshell, it demonstrates that the tumour microenvironment plays an important role in cancer spreading by synthesising and secreting growth factors and suggests that targeting growth-factor-activated Akt in combination therapy could be a valuable therapeutic approach in treating head and neck cancer patients.

**Abstract:**

This review aims to provide evidence for the role of the tumour microenvironment in cancer progression, including invasion and metastasis. The tumour microenvironment is complex and consists of tumour cells and stromal-derived cells, in addition to a modified extracellular matrix. The cellular components synthesise growth factors such as EGF, TGFα and β, VEGF, and NGF, which have been shown to initiate paracrine signalling in head and neck cancer cells by binding to cell surface receptors. One example is the phosphorylation, and hence activation, of the signalling protein Akt, which can ultimately induce oral cancer cell migration in vitro. Blocking of Akt activation by an inhibitor, MK2206, leads to a significant decrease, in vitro, of cancer-derived cell migration, visualised in both wound healing and scatter assays. Signalling pathways have therefore been popular targets for the design of chemotherapeutic agents, but drug resistance has been observed and is related to direct tumour–tumour cell communication, the tumour–extracellular matrix interface, and tumour–stromal cell interactions. Translation of this knowledge to patient care is reliant upon a comprehensive understanding of the complex relationships present in the tumour microenvironment and could ultimately lead to the design of efficacious treatment regimens such as targeted therapy or novel therapeutic combinations.

## 1. Introduction

Cells move in response to events early in the life of all developing embryos. One of the earliest migration events is collective cell migration during gastrulation [1]. During gastrulation, cells that will become epithelial cells undergo a transition in a series of events collectively known as epithelial to mesenchymal transition (EMT) and then migrate through the primitive streak. These events are largely driven by growth factors such as platelet derived growth factor (PDGF) or fibroblast growth factor (FGF) in a phosphoinositide-3-kinase (PI3K) dependent manner [2]. PI3K, a lipid kinase, is the upstream signalling molecule of the PI3K-Akt signalling pathway which has a huge role in human development and cancer. This lipid kinase activates a membrane phospholipid, phosphatidylinositol 4,5-bisphosphate (PIP2) by phosphorylation, generating phosphatidylinositol 3,4,5-trisphosphate (PIP3). PIP3 regulates a diverse set of effector proteins including small GTPases and a group of oncogenic protein kinases called Akt or protein kinase B (PKB). Akt controls a range of cellular bioactivity including, cell growth, proliferation, survival, metabolism, and migration [3].

In general, cells that are undergoing EMT migrate by one of two main mechanisms: single cell migration or collective cell migration. During single cell migration, cells migrate as individuals having no cell–cell interactions. Two different phenotypes can be displayed during the movement of single cells. These are described as either having an amoeboid or mesenchymal phenotype. Cells with an amoeboid phenotype are generally rounded in shape with a number of different variants. The mesenchymal phenotype of cells has an elongated cell body with longer protrusions. The cells differ in terms of their contractility: amoeboid showing increased contractility (under the influence of the Rho signalling), while the mesenchymal phenotype expresses low contractility [4]. The migration of single cells through a tissue uses a multi-step process occurring through a cyclical process. The first step is protrusion from the leading edge using filopodia, lamellipodia, podosomes, or invadopodia. The second step is adhesion force generation, where the cell builds up a force strong enough to pull it through the matrix. The third step involves proteolysis in focussed areas. The fourth step being contraction of the actin–myosin cytoskeleton and finally the retraction of the rear end of the cell and its release [5]. The PI3K signalling pathway has been implicated in cell migration due to its role in controlling cytoskeletal re-arrangements and the enrichment of PtdInsP3 in the leading edge membrane of several cell types during directed cell migration [6]. It appears to be roles controlling the small GTPases, Rac and Raf, that dominate downstream of PI3K, in the control of cell autonomous migration [7]. Our own data suggest a role for Akt in the migration of fibroblasts and epithelial cells [8,9]. In Chicken Embryonic Fibroblast (CEF) cells, the PI3K-Akt signalling pathway activates downstream p70S6K1 which in turn activates the Rac1 protein. Activated Rac1 is involved in actin filament remodelling, hence cell migration [10]. The PI3K-Akt pathway activates p70S6K1 in ovarian cancer, which in turn stimulates the activation of Rac1 and cdc42 and their downstream effector molecule p21 activated kinase (PAK1) [11].

Recent evidence in pancreatic ductal cancer and oesophageal squamous cell carcinoma have suggested that Akt stimulates Girdin activation which sequentially regulates actin reconstruction and cell motility [12,13].

**Figure 1:**
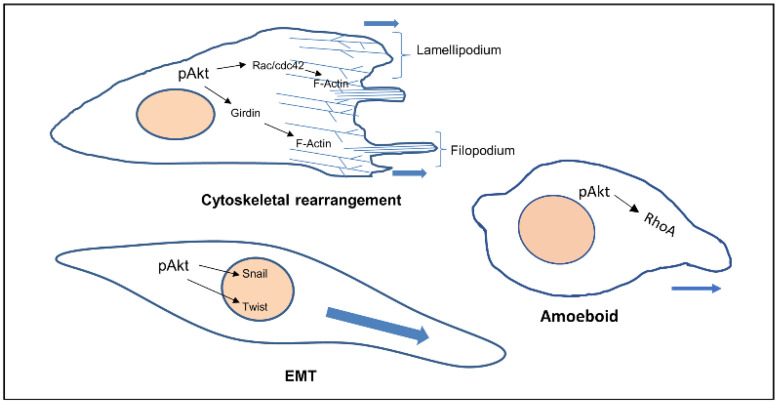
Cellular phenotypes during migration and the role of activated Akt. Activated Akt triggers the activity of downstream substrates (Rac, Girdin, Twist, RhoA) that dictate the various modes of cell migration.

Akt also phosphorylates Twist1 which promotes EMT by modulating its transcriptional target, TGFβ2. Increased TGFβ2 enhanced TGFβ receptor signalling, which in turn maintains hyperactive PI3K-Akt signalling [14]. Activated Akt was also found to inhibit DLC1 (Deleted in Liver Cancer 1) (GAP for RhoA), increasing levels of RhoA, facilitating the formation of focal adhesions, in turn increasing amoeboid type cell migration [15] (Figure 1).

Collective cell migration is the migration of cells as a group or sheet. The cells move in the same direction and at similar speed, it is slower but more efficient than single cell migration. There are two types of cells (leader and follower) in collective cell migration, based upon their relative position between cell clusters. The leader cell has the ability to sense the microenvironment, interact with the extracellular matrix (ECM), and is responsible for direction and speed of migration of the whole cluster. The follower cells have the ability to control their leader and are important in polarisation and chemotaxis. A successful coordination of the leaders and followers induces collective cell migration. In vivo and in vitro, leaders and follower cells can exchange their place and roles [16,17].

**Figure 2:**
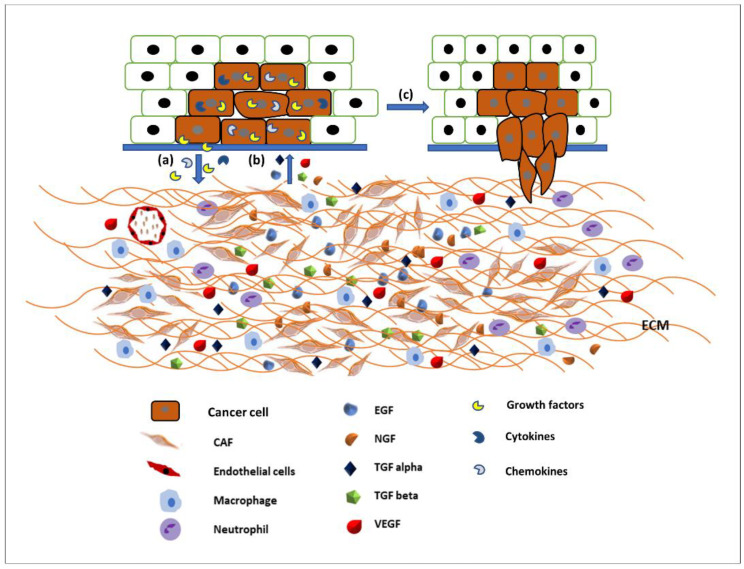
Communication between the tumour microenvironment and tumour cells. (a) Tumour cells release various growth factors, cytokines, and chemokines to recruit stromal cells, immune cells, and vascular cells. (b) Recruited cells then proliferation and overexpress growth factors such as EGF, TGFα and β, VEGF, NGF, etc., to initiate signalling pathways in tumour cells. (c) Activated signalling pathways then trigger various cellular activity such as tumour cell proliferation and migration and invasion.

The ECM is the major structural component of the tissues of the body, being mainly composed of proteins, glycoproteins, glycosaminoglycans, and polysaccharides in a network. In a normal body this matrix would be laid down by connective tissue cells, such as fibroblasts. However, this tissue is also able to host tumour cells (seed and soil theory [18]) and control whether the tumour will be able to grow and metastasise. Some tissues may be able to block the ability of tumour cells moving freely through a tissue [18,19]. Therefore, the modulation of the ECM is essential for the migration of tumour cells [20]. The link between the migration of cancer cells and the spread of cancer has been established for many years and an overview produced earlier this year [21]. The original Hallmarks of Cancer included the phrase ‘tumour invasion and metastasis’ [22]. The concept of the Hallmarks of Cancer allowed new discussion around older theories, which had fallen out of mainstream thinking such as the tumour microenvironment [23]. Therefore, it is not just the cancer cells themselves that are important, it is the interactions of the tumour and the stroma that are vital. Studies into these interactions between tumour and “host” have been integral to investigating the role of growth factors (and their receptors), extracellular matrix molecules (and their receptors), and cell signalling pathways and the crosstalk between all of these factors. The tumour microenvironment (TME) is made up of a complex mixture of tumour cells and stromal-derived cells, such as cancer associated fibroblasts (CAFs), immune cells, and endothelial cells, in addition to a modified ECM. Growth factors and ECM both modulate cell signalling pathways. Human tumours are more than a mass of accumulating malignant cancer cells. Tumour cells can efficiently recruit stromal cells, immune cells, and vascular cells by secreting stimulatory growth factors, chemokines, and cytokines. These recruited cells then overexpress and release growth factors and intermediate metabolites, as well as reorganising the tissue structure to build the microenvironment. The shared communication between cancer cells and the microenvironment eventually leads to enhanced proliferation and metastasis [24] (Figure 2).

Therefore, the aim of this review is to provide evidence for the role of the tumour microenvironment in synthesising and secreting growth factors and to appraise the data of different growth factors found to increase head and neck cancer cell motility by activating the PI3K-Akt signalling pathway in terms of single and collective cell migration. The data provided here is a comparison of a normal and a cancer cell line in response to the following growth factors, EGF, TGFα, TGFβ-1, VEGF, and NGF, and a specific inhibitor of Akt, MK2206.

## 2. Epidermal Growth Factor (EGF)

Epidermal growth factor (EGF) was first identified by Dr Stanley Cohen from mouse salivary gland extracts while working with Dr Rita Levi-Montalcini in Washington University, St. Louis [25]. In 1962, Cohen reported the isolation of a polypeptide from the submaxillary gland of male mice that accelerated eyelid opening and tooth eruption in the newborn animal, later identified as epidermal growth factor (EGF) [26]. Dr Levi-Montalcini and Cohen were awarded the 1986 Nobel Prize in Physiology or Medicine for the discovery of the first growth factors, NGF (nerve growth factor) and EGF [27]. EGF, one of the most abundant growth factors in the tumour microenvironment [28], can be produced by cancer cells and non-cancerous cells such as endothelial cells [29], mesenchymal stromal cells [30], and macrophages [31,32]. EGF binds with the epidermal growth factor receptor (EGFR) which belongs to the ErbB family of receptor tyrosine kinases (RTKs), which includes four members: EGFR, ErbB-2, ErbB-3, and ErbB-4 [33]. It is well recognised that upon ligand binding, the EGFR signalling pathways facilitate a wide range of cellular responses such as proliferation, differentiation, migration, and survival. Moreover, amplified expression of EGFR or its ligands, or both, are found in a majority of human carcinomas [34]. EGF-like growth factors can be produced either by the same cells that express EGFRs in an autocrine manner or by the surrounding cells (including stromal cells) in a paracrine manner [35]. Therefore, sustained activation of EGFR signalling in non-malignant cells in the tumour microenvironment might influence the behaviour of transformed cells and can play an important role in tumour progression. There is evidence to suggest that overexpression of the EGFR protein can be triggered by either gene mutation or by tumour hypoxia, which is one of the common traits in the tumour microenvironment [36,37]. Meanwhile, the EGFR system is believed to be involved in tumour metastases as well as angiogenesis, which are two important phenomena that promote tumour progression [38,39]. Moreover, it was evident that tumour hypoxia was required to upregulate EGFR protein expression in hypoxic cancer cells. These findings reveal an important link between tumour hypoxia and upregulation of the EGFR in the majority of human cancers that do not display genetic alterations of the receptor [40].

**Figure 3:**
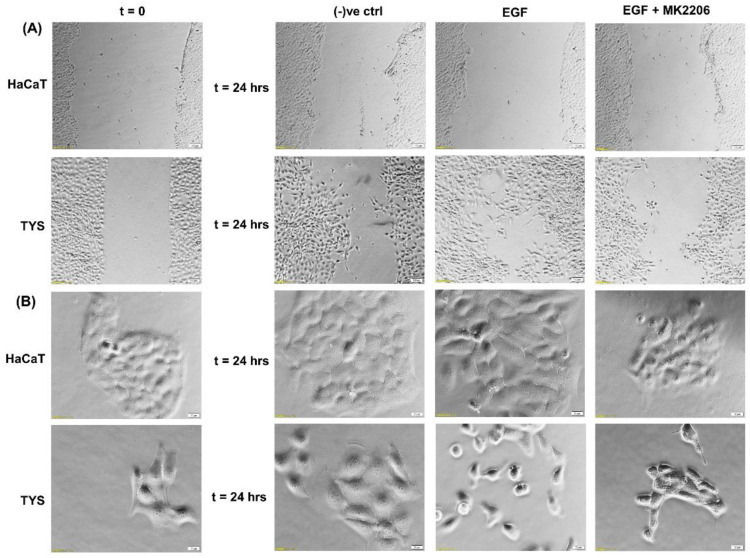
Effect of EGF on oral cancer cell migration. (**A**) A wound was created manually on the cell monolayer as previously described [61] and treated with the EGF with or without the inhibitor for 24 h. EGF stimulated oral adenoid cancer cells (TYS) to close the gap in the wound healing assay (collective cell migration) compared to the negative control. The Akt inhibitor, MK2206, effectively blocked EGF-induced oral cancer cell migration. Normal keratinocytes (HaCaT) were not stimulated to close the gap in response to EGF. Serum-free medium was used as the negative control. Images were captured at t = 0 and at t = 24 h at 50× magnification. (**B**) Cells were plated at low density to form small colonies of cells for the scatter assay as previously described [61] and were then treated with the test conditions for 24 h. EGF stimulated TYS cells to scatter away from the cell colonies (single cell migration) and MK2206 blocked EGF-induced scattering of oral cancer cells. HaCaT did not stimulate scattering in response to EGF. Serum-free medium was used as the negative control. Images were captured at t = 0 and at t = 24 h at 200× magnification. An inverted microscope (Olympus IX70, Tokyo, Japan) with a brightfield camera (SC50) was used to capture the images and CellSense software (Olympus) was used to process the images. The experiments were repeated more than 3 times.

Although EGFR is expressed in the normal oral epithelium as well as in the majority of OSCC cells [41,42], it is also a therapeutic target for the treatment of oral cancer. EGFR biomarker detection in oral squamous cell carcinoma may fulfil multiple roles in cancer diagnostics, not only for early detection, but also at diagnosis for prognostic evaluation and treatment [43,44]. Researchers also found an overexpression of EGFR in the plasma membrane correlated with poor prognosis in tongue cancer [45,46]. More than 80% of invasive head and neck squamous cell carcinomas overexpressed EGFR, which is often linked to an unfavourable clinical outcome, high recurrence, and low survival rates [47,48]. Activation of EGFR signalling is associated with a malignant phenotype, manifested by angiogenesis, inhibition of apoptosis, as well as an increased metastatic potential [49]. Studies using various cell types have demonstrated that the downstream signalling pathways of EGFR are involved in the regulation of cell motility [50,51]. Certain mitogen-activated protein (MAP) kinases, including extracellular-regulated kinase (ERK), Jun kinase, and tumour protein (p38), are able to affect various cell functions, including migration [52]. Phosphatidylinositol-3 kinase (PI3K) controls cell motility through the activation of protein kinase B (Akt) and other targets [53,54]; however, cell-dependent differences in these regulatory mechanisms exist [52,55,56,57]. Phosphorylated Akt (pAkt) at S473 and T308 is an activated type of Akt, which plays a major role in various human cancers. Many studies have identified that Akt may be a potential oncogene in humans and over-expression of phosphorylated Akt (pAkt) has been identified in various cancers, including oral cancer [58]. Akt activation is linked with increased cancer cell invasion in various cancers, including oral squamous cell carcinomas. Hence, pAkt may be a good predictor of cancer aggressiveness [47,59,60]. Data from our laboratory showed that EGF stimulated oral adenoid cancer cell (TYS) migration in both the wound healing and scatter assay and the pAkt inhibitor, MK2206, effectively blocked TYS cell migration, but normal keratinocytes were not stimulated to migrate (Figure 3). Detailed experimental methods are described in the Appendix A.

Chang et al. in 2013 demonstrated the role of pAkt and EGFR variant III in oral carcinomas, acting as determinant factors for patient survival; which could be used as prognostic biomarkers [47]. The abundance of EGF together with the overexpression of EGFR observed in diverse types of cancers, including head and neck cancer [29,62,63], lead to activation of the downstream signalling pathways that facilitate the EMT process [61,64], which consequently renders cancer cells with plastic properties and increased invasive and metastatic capabilities [29,64,65]. The activation of the EGF/EGFR signalling pathways facilitates the EMT process and enrichment of ALDH+/CD44 high cancer stem cells (CSCs)-like cells with increased invasiveness/metastasis potentials both in vitro and in vivo [64]. These findings, together with previous studies [22,34], support the notion that the EMT process and acquisition of CSC-like phenotypes is driven by activated EGF/EGFR signalling cascades, which may play a critical role in the metastasis and recurrence of OSCC [29,64,65].

## 3. Transforming Growth Factor α (TGFα)

A new polypeptide called Sarcoma Growth Factor (SGF) was first discovered in retrovirus-transformed rat kidney fibroblasts cells, but soon it was apparent that this factor was a mixture of two distinct substances called TGF (transforming growth factor) α and β [66,67]. TGFα is widely expressed in both normal epithelium and tumour cells and it is considered a ligand for EGFR [68]. TGFα is often expressed by mesenchymal cells, in the gastrointestinal tract, lung, liver, kidney, mammary gland, dermis, gonads, skeletal muscle, and nerve cells within the central and peripheral systems. Although TGFα commonly acts via autocrine or paracrine signalling in solid tissues, it can also mediate paracrine signalling by activated macrophages, monocytes, neutrophils, and eosinophils [69,70,71]. TGFα is structurally similar, but more potent than EGF and showed similar effects in a number of assays [68]. Both EGF and TGFα have six cysteine residues in the similar positions and have 35% sequence homology [72]. They bind to the same receptor, EGFR, due to the location of the three disulfide bridges [73].

Research provided evidence that mRNA and protein levels of TGFα and EGFR increased in oral cancer patients in comparison to disease-free controls and poor prognosis might be related to this elevation level of TGFα and EGFR [74,75]. mRNA levels of TGFα and EGFR also increased in the normal mucosa of oral cancer patients, suggesting that TGFα/EGFR gene transcription is an early event of carcinogenesis or could be a foundation to a tumour [74,76]. TGFα and EGFR also been reported to play important role in the proliferation of oral cancer cells but not in the proliferation of normal epithelium [77]. Elevated levels of TGFα in premalignant, dysplastic, and HNSCC patients suggests that upregulation of TGFα represents an early event in HNSCC pathogenesis [74,76,77].

**Figure 4:**
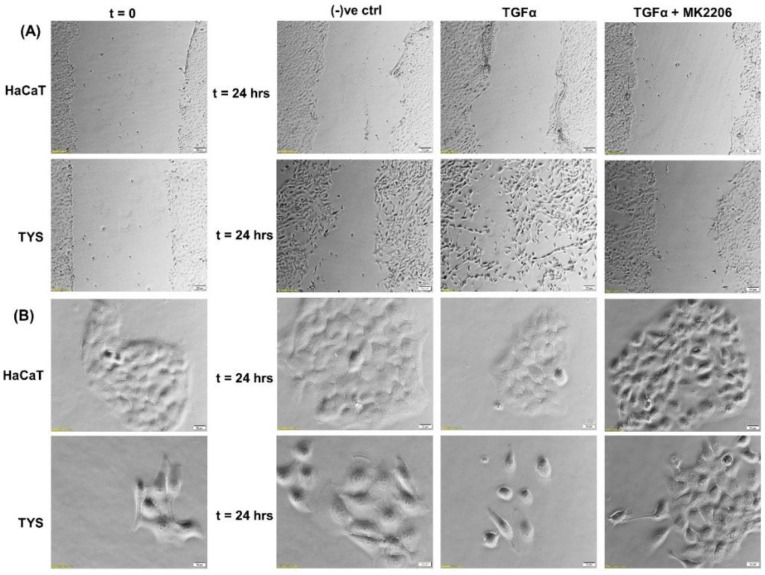
Effect of TGFα on the migration of oral cancer cells. (**A**) A wound was created manually on the cell monolayer and treated with TGFα with or without the inhibitor for 24 h. TGFα stimulated the oral adenoid cancer cells (TYS) to close the gap by migration in the wound healing assay, in comparison to the negative control where little or no migration was observed. The Akt inhibitor, MK2206, effectively blocked TGFα-induced oral cancer cell migration. Normal keratinocytes (HaCaT) were not stimulated to migrate in response to TGFα. Serum-free medium was used as the negative control. Images were captured at t = 0 and at t = 24 h at 50× magnification. (**B**) Cells were plated at low density to form small colonies of cells for the scatter assay and were then treated with the test conditions for 24 h. TGFα stimulated TYS cells to scatter or migrate away from the compact colonies and a mesenchymal-like morphology of the scattered cells was observed. MK2206 partially blocked TGFα-induced scattering of oral cancer cells. HaCaT did not stimulate cell scattering in response to TGFα. Serum-free medium was used as the negative control. Images were captured at t = 0 and 24 h at 200× magnification. An inverted microscope (Olympus IX70, Tokyo, Japan) with a brightfield camera (SC50) was used to capture the images and CellSense software (Olympus) was used to process the images. The experiments were carried out more than 3 times.

The biological importance of this growth factor, in head and neck cancer progression, is supported by the findings demonstrating that survival of HNSCC patients correlates significantly with TGFα protein expression levels in the primary tumour, independent of other clinical and pathological parameters including the presence of regional metastases (N-stage) [75,78]. EGFR activation following TGFα binding results in stimulation of proliferative and pro-survival intracellular signalling, through the mitogen-activated protein kinase (MAPKs) cascade, PI3K/Akt/mTOR and JAK/STAT pathways [79,80]. In vitro studies using the oral adeno squamous cell carcinoma cell line (TYS), in our laboratory, showed stimulation of migration in response to TGFα in both wound healing and scatter assays. The blocking effect of MK2206, a pAkt inhibitor, suggested that TGFα-stimulated oral cancer cell migration might be Akt signalling pathway-dependent (Figure 4).

## 4. Transforming Growth Factor β (TGFβ)

Transforming growth factor (TGF) β was first isolated and characterised by Roberts and Sporn, as a secreted polypeptide capable of inducing fibroblast growth and collagen production [81]. TGFβ regulates a variety of biological functions such as growth, development, tissue homeostasis, and regulation of the immune system [82,83]. Soon after its discovery, a dual role of TGFβ was recognised, as it was also found to inhibit cell proliferation [84,85]. Because of its prominent role in the regulation of cell growth, differentiation, and migration, TGFβ is considered essential for cancer progression. TGFβ functions as a tumour suppressor during the early stages of tumourigenesis. However, tumour cells lose their growth inhibition response to TGFβ as tumours progress and may instead respond by initiating EMT and by stimulating cell migration [86]. Three isoforms of TGFβ (TGFβ 1, TGFβ 2, and TGFβ 3) instigate cellular phenotypical changes through canonical (SMAD) and non-canonical (MAPK, JAK/STAT, and PI3K/Akt) signalling pathways that mediate its role both as a tumour suppressor and a tumour promoter. Three types of TGFβ receptors (RTKs) are responsible for initiating both canonical and non-canonical signalling; TGFβRI, II, and III [87,88]. Experimental data support the idea that both loss and gain of TGFβ/TGFβR-mediated signalling is pro-tumourigenic, as the overexpression of TGFβ and downregulation of signalling results in increased tumour metastasis. It is now appreciated that the effects of TGFβ signalling in cancer cells extends beyond cancer-cell-autonomous mechanisms into the tumour microenvironment and this is essential for tumour progression [89,90,91]. In pre-malignant tumours, TGFβ is secreted into the microenvironment initially to control proliferation and cancer progression, but it is ultimately utilised by cancer cells to stimulate their malignant properties. Secretion of TGFβ from cancer cells regulates their own properties within the tumour mass in an autocrine and paracrine fashion. However, infiltrating stromal cells including fibroblasts, leukocytes, macrophages, bone-marrow-derived endothelial cells are other sources of TGFβ [88,92]. A primary role of TGFβ in regulating the tumour microenvironment, is its contribution to the conversion of fibroblasts to myofibroblasts, also known as cancer-associated fibroblasts (CAFs) [93,94]. CAFs upregulate ECM proteins and lead to fibrosis, matrix stiffening, and desmoplasia. Increased stiffening of the matrix, in turn, increases the compressive forces developed inside a tumour (due to its growth in the confined space of the host tissue) and contributes to further activation of TGFβ from the ECM [95,96]. Increased TGFβ in cancer cells also increases ECM deposition and endothelial cell recruitment and proliferation. These microenvironmental changes endorse epithelial and stromal cell phenotypical responses, which subsequently affect tumour progression [86].

**Figure 5:**
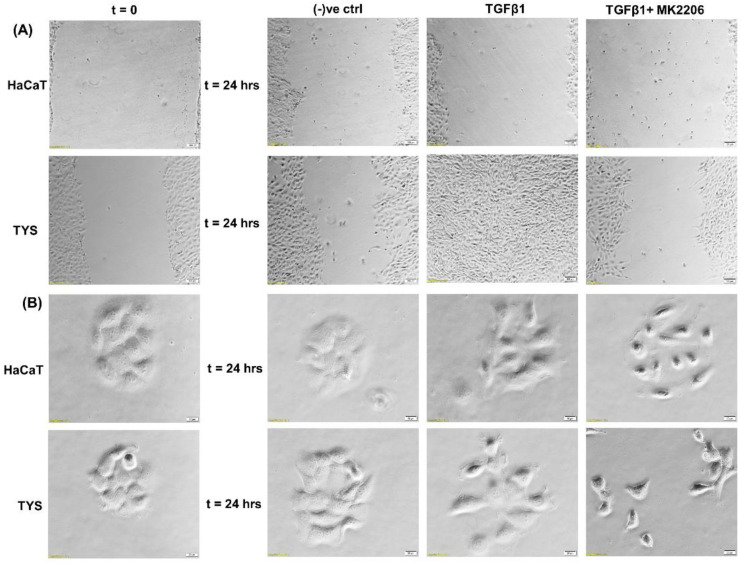
Oral cancer cell migration in response to TGFβ. (**A**) TGFβ with or without the inhibitor was used in the wound healing assay for 24 h. TGFβ-stimulated TYS cell migration in the wound healing assay was compared to the negative control. The Akt inhibitor, MK2206, effectively blocked TGFβ-induced oral cancer cell migration. Normal keratinocytes (HaCaT) were not stimulated to migrate in response to TGFβ. Serum-free medium was used as the negative control. Images were captured at t = 0 and 24 h at 50× magnification. (**B**) Cells were plated at low density to form small colonies of cells for the scatter assay and were then treated with the test conditions for 24 h. TGFβ stimulated TYS cells to migrate away from the compact colonies. However, MK2206 did not block TGFβ-induced scattering of oral cancer cells. HaCaT cells were not stimulated to scatter in response to exogenous TGFβ. Serum-free medium was used as the negative control. Images were captured at t = 0 and 24 h at 200× magnification. An inverted microscope (Olympus IX70, Tokyo, Japan) with a brightfield camera (SC50) was used to capture the images and CellSense software (Olympus) was used to process the images. The experiments were carried out more than 3 times.

Reports suggest that TGFβ1 is overexpressed in around 80% of human head and neck cancer patients and correlates with more advanced disease and reduced survival [97,98]. Mutations of TGFβRII have been reported in 21% of oral squamous cell carcinomas [98]. Deregulated TGFβ signalling is associated with poor prognosis, partly due to the induction of EMT. In OSCC, TGFβ signalling has been implicated in EMT through Snail and upregulation of matrix metalloproteinase-9 (MMP9) [99,100]. It has also been reported that TGFβ1 may enhance EMT after a long-term co-stimulation with EGF compared to TGFβ1 or EGF alone; markedly enhancing OSCC invasiveness [101]. Furthermore, CAFs increased matrix stiffness through activation of MMPs and YAP1, consequently facilitating invasion in OSCC [102]. In a recent study, we showed that CAFs stimulate the migration of oral cancer cells via PI3K/Akt signalling pathways [103]. We also report here that TGFβ stimulated TYS to migrate in the wound healing assay by an Akt-dependent manner. Normal keratinocytes were not stimulated to migrate in response to exogenous TGFβ1 (Figure 5).

It was also evident that TGFβ can augment the proliferation of CSCs in OSCC, via the Akt signalling pathway [104].

## 5. Vascular Endothelial Growth Factor (VEGF)

In 1971, Judah Folkman first reported a polypeptide secreted by tumours causing angiogenesis and named it tumour angiogenesis factor [105]. Senger et al. then described a factor in 1983 called vascular permeability factor secreted by tumours in hamsters and guinea pigs [106]. Ferrara and Henzel then purified and cloned an identical factor in 1989. They named this growth factor vascular endothelial growth factor (VEGF) based on its mitogenic effect on adrenal-cortex-derived capillary endothelial cells [107]. VEGF is synthesised by various cell types including macrophages [108], platelets [109], keratinocytes [110], renal mesangial [111], and tumour cells [112,113]. The VEGF family of genes is composed of at least 7 members, including VEGFA, VEGF B, VEGF C, VEGF D, and VEGF E which bind mostly with VEGF receptors 1/2/3, types of RTKs. VEGFA binds with VEGFR 1 and 2 inducing both physiological and pathological angiogenesis, including tumour angiogenesis. VEGF A has several isoforms, most commonly named as VEGF121, VEGF165, VEGF189, and VEGF206 and can be soluble or matrix-bound [114]. The interaction of matrix proteins with VEGF is considered significant for the angiogenic switch, enabling the transition from hyperplasia to malignant tumour formation [115]. Matrix-bound VEGF elicits prolonged activation of VEGFR2 in endothelial cells associating with integrin β1, resulting in the extended activation of downstream signalling pathways. This response is lacking upon exposure to soluble VEGF [116]. Multiple downstream signalling pathways are activated upon VEGF isoforms binding with their cognate membrane-bound receptors. VEGF signalling pathways include the Ras/MAPK signalling pathway which regulates cell proliferation and gene expression, the FAK/Paxillin pathway involved in the cytoskeletal rearrangement, the PI3K/Akt pathway regulating cell survival and migration, and the PLCγ pathway controlling vascular permeability [117].

It is now established that angiogenesis is crucial for the proliferation and metastasis of solid tumours including head and neck cancers. VEGFRs have shown to be present in the tumour cells and tumour microenvironments including endothelial cells. Autocrine and paracrine signalling mediated interaction happened between tumour cells and their microenvironments that stimulates angiogenesis, uncontrolled cellular growth, and metastasis [118]. Assessment of VEGF and associated receptors thus became a dependable prognostic tool in HNSCCs, predicting metastasis and poor survival. Study have shown that VEGF-positive oral cancer cases range from 25 to 100% with a mean positivity of around 78% [119]. VEGF overexpression is linked to the poor survival rates [120,121,122]. We reported that VEGF stimulated the migration of oral-cancer-associated fibroblasts (CAFs) which remodel the extracellular matrix and stimulate EMT of oral cancer cells by secreting various growth factors. Thus, VEGF plays a significant role in invasion and metastasis.

**Figure 6:**
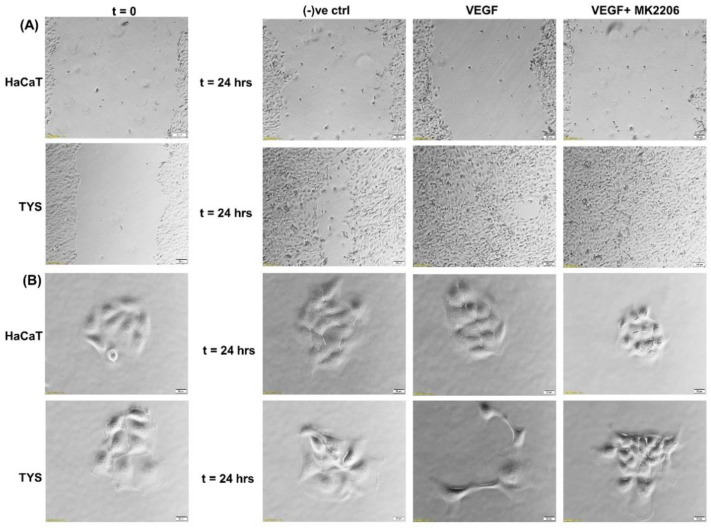
Oral cancer cell migration in response to VEGF. (**A**) VEGF ± inhibitor was used in the wound healing assay for 24 h. VEGF stimulated TYS migration in the wound healing assay in comparison to the negative control. The Akt inhibitor, MK2206, effectively blocked VEGF-induced oral cancer cell migration. HaCaT cells were not stimulated to migrate in response to VEGF. Serum-free medium was used as the negative control. Images were captured at t = 0 and 24 h at 50× magnification. (**B**) Test conditions were applied in the scatter assay for 24 h. VEGF stimulated TYS cells to migrate away from the compact colonies and a mesenchymal-like cell morphology was observed in the scattered cells. MK2206 effectively blocked VEGF-induced scattering of oral cancer cells. HaCaT cells were not stimulated to scatter in response to VEGF. Serum-free medium was used as the negative control. Images were captured at t = 0 and 24 h at 200× magnification. An inverted microscope (Olympus IX70, Tokyo, Japan) with a brightfield camera (SC50) was used to capture the images and CellSense software (Olympus) was used to process the images. The experiments were carried out more than 3 times.

The VEGF-induced migration of CAFs and oral cancer cells was PI3K/Akt signalling pathway-dependent [9,103]. Figure 6 shows that TYS are stimulated to migrate and display mesenchymal-like phenotype in response to exogenous VEGF. VEGF-induced TYS cell migration was then effectively blocked by the Akt inhibitor. Our data also suggested that Akt, phosphorylated at T308, is a reliable biomarker in VEGF positive smoking and alcohol induced HNSCC progression; however VEGF-induced Akt phosphorylation at S473 might be a prognostic biomarker in HNSCC [123].

## 6. Nerve Growth Factor (NGF)

NGF is a neurotrophic factor which was the first growth factor discovered in cancer biology by Levi-Montalcini in the 1940s, as a substance secreted by a mouse sarcoma in 3-day-old chick embryos, that stimulated neurite outgrowth and neural survival [124]. NGF is produced by the central and peripheral nervous system and immune cells. The largest amount of the neurotrophin is produced in the submaxillary glands [125].

NGF is a member of the neurotrophins family, which also includes brain-derived neurotrophic factor (BDNF), neurotrophin-3 (NT-3), and NT-4/5 [126]. The history of NGF and other neurotrophic factor research is strongly associated with the field of neuroscience, where NGF promotes survival, differentiation, and functional activity of peripheral sensory and sympathetic nerve cells [127]. There are non-neural functions of NGF outside its classical duty in the peripheral and central nervous system, for example, roles in the development of the reproductive system, the endocrine, cardiovascular, and immune systems [128]. Moreover, NGF has protective and treatment roles in Alzheimer’s disease [129], corneal ulcers, and glaucoma [130]. Recent progress in NGF research has shown that NGF has a role in carcinogenesis and the pathogenesis of many tumours by regulating cell proliferation, invasion, and therefore cancer cell survival [131]. These various actions of NGF are dependent on cell type and the presence of NGF receptors in the cancer cells.

Neurotrophins mediate their functions through two structurally distinct classes of transmembrane receptor tyrosine kinases; low-affinity receptor, the p75 neurotrophin receptor (p75^NTR^) which binds all of the neurotrophins with approximately equal affinity, activating nuclear factor-kappa B (NF-κB) and c-Jun N-terminal kinase (JNK) leading to cell survival and death, respectively. Specific high-affinity tyrosine kinase receptors called tropomyosin related kinases (Trks) exhibit specificity in neurotrophin binding, leading to proliferation, survival, or cell death via the activation of PI3K/Akt, Ras/MAPK, and PLCγ pathways [132]. In total, 17.4% OSCC cases have shown perineural invasion (PNI) with overexpression of nerve growth factor and tyrosine kinase A in 84% and 92% cases, respectively. Frequency of nerve growth factor and tyrosine kinase A overexpression were significantly higher in the tumours with PNI compared to the tumours without PNI. PNI is also linked to the late stage tumours and poor survival [133].

**Figure 7:**
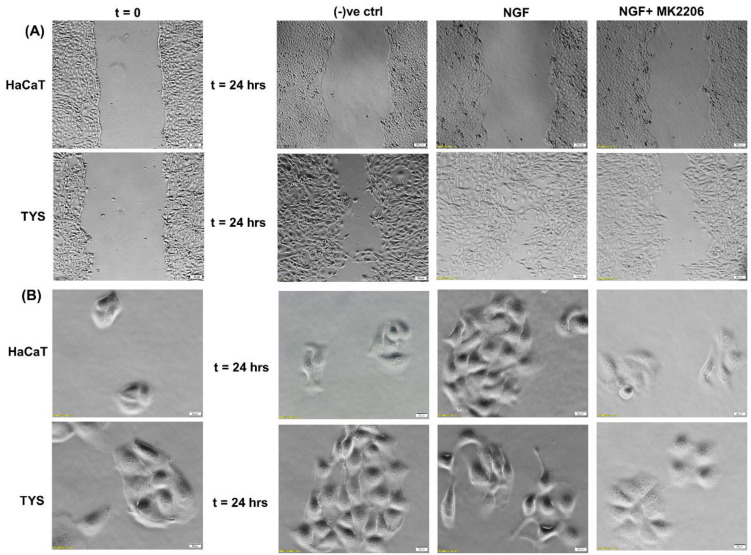
Effect of NGF on oral cancer cell migration. (**A**) NGF with or without the inhibitor was used in the wound healing assay for 24 h. NGF stimulated higher numbers of TYS to migrate in the wound healing assay in comparison to the negative control. The Akt inhibitor, MK2206, effectively blocked TGFβ-induced oral cancer cell migration. Normal keratinocytes (HaCaT) were not stimulated to migrate in response to TGFβ. Serum-free medium was used as the negative control. Images were captured at t = 0 and 24 h at 50× magnification. (**B**) Cells were plated at low densities to form small colonies for the scatter assay and were then treated with the test conditions for 24 h. NGF stimulated TYS cells to migrate away from the compact colonies and a mesenchymal-like cell morphology was observed in the scattered cells. MK2206 effectively blocked NGF-induced scattering of the oral cancer cells. HaCaT cells were not stimulated to scatter in response to NGF. Serum-free medium was used as the negative control. Images were captured at t = 0 and 24 h at 200× magnification. An inverted microscope (Olympus IX70, Tokyo, Japan) with a brightfield camera (SC50) was used to capture the images and CellSense software (Olympus) was used to process the images. The experiments were carried out more than 3 times.

NGF has been shown to play a role in tumour proliferation and perineural spread (PNS) in oral cancer [134] and it has been shown that oral squamous cell carcinoma (OSCC) with evidence of PNS has an increased expression of NGF and TrkA and this data suggested that NGF and TrkA are correlated with the development of PNS [134]^.^. Another study also suggested that NGF can stimulate the adhesion and migration of salivary adenoid cystic carcinoma (AdCC) cells and therefore facilitate the invasion of the AdCC to the nerves [135]. A higher expression of NGF protein was found in tissues of OSCC than in normal oral epithelium [136] and in the tissues of AdCC [137]. NGF has shown to play a role in tumour proliferation and in perineural invasion in ex vivo and in vitro studies where a tissue of OSCC and human oral cancer cells has been used to investigate the expression of NGF and the concentration of NGF protein, respectively, and the results showed that the tissue biopsies from OSCC showed a strong NGF immunoreactivity and that NGF protein concentration was higher in oral cancer cells than in normal cells [138]. A study by Søland et al. (2008) was conducted to evaluate the prognostic significance of p75^NTR^ and the expression of p75^NTR^ in 53 paraffin-embedded archival tissues of OSCC for the first time, both at the invasive front and in the rest of the tumour tissue and reported that p75(NTR) was expressed in all OSCCs, and p75^NTR^ expression and the pattern of invasion were significantly associated with poor prognosis in OSCC [139]. Thus, NGF can stimulate morphological differentiation, adhesion, proliferation, and migration in OSCC and therefore enhance perineural invasion and possibly facilitate metastasis. A recent study from our group also reported that Akt is phosphorylated at both residues by NGF controlling OSCC cell migration and, furthermore, the addition of an Akt pathway inhibitor blocks the NGF-induced OSCC migration and therefore invasion [140] (Figure 7).

## 7. Discussion and Conclusions

Cetuximab, a monoclonal antibody, targeting EGFR is the only FDA-approved targeted therapy for the treatment of head and neck squamous cell carcinoma, in combination with radiation therapy or as a single agent in patients who have had prior platinum-based therapy. The response rate, as a single agent, is only 13% and the patients who respond initially eventually develop resistance [141,142]. It was hypothesised that acquired cetuximab resistance in HNSCC may result from the activation of compensatory signalling pathways following cetuximab treatment. These compensatory signalling pathways can withdraw the inhibitory effects of cetuximab through phosphorylation of key proteins, thereby promoting cell survival [143]. A protein phosphorylation profiling study showed increased phosphorylation of Akt after cetuximab treatment in acquired cetuximab-resistant cells compared to cetuximab-sensitive cells. Additionally, the study observed an additive to synergistic interaction between cetuximab and the Akt inhibitor, MK2206 in cetuximab-sensitive and acquired cetuximab-resistant HNSCC cell lines [143]. Montagut et al. showed that an EGFR mutation at S492R inhibits Cetuximab binding with the receptor but does not block EGF or TGFα binding. EGF or TGFα may therefore activate the downstream PI3K/Akt signalling pathway. Cetuximab resistance can also be mediated by the activation of the Akt signalling pathway in an alternative way, such as the overexpression of other growth factors (TGFβ, VEGF, NGF) and their associated receptors by the tumour cells and/or the tumour microenvironment [142]. Few recent clinical trials using Akt inhibitor alone to treat late stage or recurrent head and neck cancer did not show a promising outcome (Table 1).

Clinical trials containing MK2206 alone observed 30–60% grade 3 toxicities in HNSCC patients [144,145,146]. However, one recently completed clinical trial (NCT01816984) observed that the combination of PI3K inhibitor, BKM120, and cetuximab caused only a few grade 3 adverse events; acute kidney injury (16.67%), electrolyte abnormality and anaemia (8.33%), and orthostatis (8.33%) with 8–9% response rate in recurrent/metastatic head and neck cancer patients [148]. Thus, this trial showed the promise in the combination therapy containing an EGFR inhibitor and PI3k/Akt inhibitor in HNSCC in terms of reducing toxicity.

**Figure 8:**
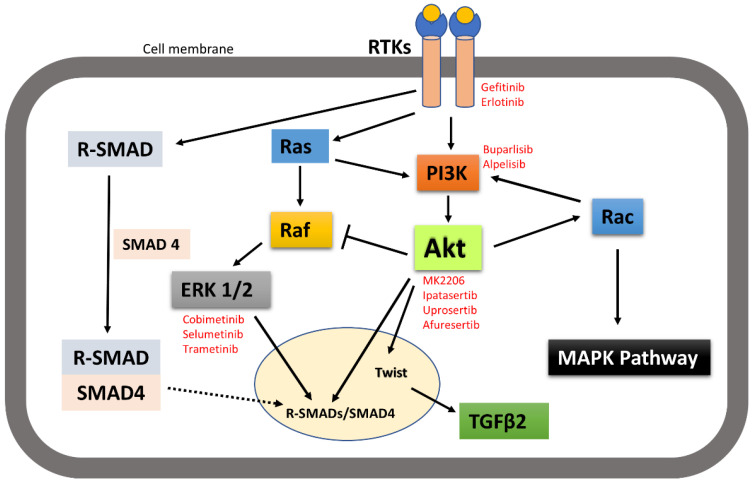
A simplistic graphical representation of the crosstalk between PI3K-Akt and RTK-activated other pathways. PI3K-Akt can crosstalk with MAPK and SMAD pathways to regulate their activities; thus, control cellular function. Solid arrow indicates activation, block arrow indicates inhibition, and dashed arrow indicates cellular distribution. Red texts indicate the inhibitors of the corresponding signalling molecules and receptors that are in various phases of clinical trial in treating cancer.

It is worth noting here that activated receptor tyrosine kinases activate not only the PI3K-Akt signalling pathway, but also other pathways including MAPK and SMAD pathways. Signalling pathways are activated in a context-dependent manner and crosstalk among each other. Hence, targeted inhibition of one pathway downstream of receptors may not affect other pathways and that adds complexity to therapeutic targeting. For example, broad crosstalk exists between PI3K-Akt and Ras-Raf-MAPK pathways. Binding of ligands such as growth factors and cytokines to RTKs activates Ras. Activated Ras in turn triggers MAPK/ERK signalling pathways. Activated Ras also recruits the p110 subunit of PI3K, which in turn activates Akt signalling pathways [149,150]. Evidence also suggests that Akt can directly inhibit Raf activity by phosphorylation, hence inhibiting the MAPK pathway [151]. It has also been suggested by IP C et al. that activated Akt can trigger Rac activity by activating p70S6K [10]. Activated Rac then further activates PI3K and MAPK and acts as a bridge between PI3k-Akt and MAPK pathway crosstalk [149]. MAPKs and Akt were also found to bind and/or phosphorylate R-SMADs to control their intracellular distribution and transcriptional activity. MAPKs and Akt also phosphorylate and regulate a variety of SMAD binding partners in the nucleus, indirectly affecting the SMAD transcriptional-activation activity [152]. Other research also suggested that the PI3K-Akt signalling pathway promotes tumour metastasis by phosphorylating Twist1, via a crosstalk between Akt and TGFβ signalling [14] (Figure 8). However, crosstalk between the PI3K-Akt pathway and other receptor tyrosine-kinase-activated signalling pathways in head and neck cancer metastasis still need to be investigated.

Published data from our research group suggested that receptor tyrosine kinase inhibitors such as Gefitinib and Erlotinib inhibited the migration of head and neck cancer by inhibiting both Akt and MAPK phosphorylation [153]. Therefore, carefully designing a clinical study using a combination of an Akt inhibitor and another signalling molecule inhibitor or receptor inhibitor in the stage I–III HNSCC patient might result in an expected positive outcome.

Metastasis is a complex and multistep process in which interactions between the cellular and structural components of the TME allow cancer cells to become invasive and spread from the primary site to a distant location. Growing evidence supports the important role of the tumour microenvironment in drug resistance, as it is the main reason for the metastasis, relapse, and incurability of various cancers. Tumour-associated macrophages exert tumour growth and survival functions; CAFs reorganise the extracellular matrix creating migration-guiding tracks for cancer cells and mesenchymal cells synthesise exosomes that increase the migratory ability of the cancer cells. TME-derived exosomes are involved in the multistep process of carcinogenesis. Exosomes act as the communication channels, encouraging crosstalk between cancer and non-cancerous cells. They are associated with the increased invasiveness and drug resistance in head and neck cancer, indicative of an attractive therapeutic target [154]. Activation of the Akt pathway is one of the mechanisms involved in resistance to radiotherapy, an effective treatment modality for HNSCC [155]. Mutschelknaus et al. (2017) showed evidence that exosomes from irradiated head and neck cancer cells enhanced Akt signalling, showed increased migratory phenotype and treatment resistance compared to non-irradiated cells [156]. Thus, targeting Akt can also be an effective therapeutic strategy in exosome-mediated radiation resistant HNSCC. Akt activation is also required for T cell activation. However, evidence has shown that the sustained activation of Akt gradually drives T cells toward terminal differentiation and weakened anti-tumour activity [157]. Crompton et al. (2014) and van der Waart et al. (2014) have shown that the inhibition of Akt signalling pathway promotes generation of potent tumour-infiltrating lymphocytes (TIL) and superior tumour reactive CD8^+^ T cells with stem cell-like properties [158,159]. This higher proliferation capacity observed in Akt-inhibited T cells could be due to the combination of increased expression of cytokine receptors such as IL-7Rα, greater expression of co-stimulatory molecules such as CD28, and less replicative senescence characteristics [159]. Urak R et al. (2017) also revealed that Akt inhibition during ex vivo expansion did not inhibit CD19CAR (chimeric antigen receptor) T cell proliferation and effector functions [160]. Adoptive transfer of ex vivo Akt-inhibited tumour reactive CD8^+^ T cells and CD19CAR T cells results in increased anti-tumour effects [158,159,160].

Direct tumour–tumour cell communication, tumour–ECM interface, and tumour–stromal cell communication contributes to drug resistance. Moreover, growth factors produced in the TME provide additional signals such as Akt for cell growth, migration, and invasion and hence metastasis [161,162]. In summary, understanding the role of the tumour microenvironment in terms of activating the Akt signalling pathway and their crosstalk in metastasis in head and neck cancer, can clearly lead to the development of more effective targeted therapies, novel therapeutic combinations, or both.

## Figures and Tables

**Table 1:** Recent clinical trials of Akt inhibitors in HNSCC patients. CR—complete response; PR—partial response; NPC—nasopharyngeal cancer; HNSCC—head and neck squamous cell arcinoma; ADCC—adenoid cystic carcinoma.

Trial Identifier	Phase	Stage of Cancer	Name of the Inhibitor	Combination	Status	Result	Ref.
NCT01349933	II	Stage IV/recurrent NPC	MK2206	None	Completed	CR–0% PR-4.8% Stable disease-52.4%	[144]
NCT01370070	II	Recurrent NPC	MK2206	None	Completed	CR–0% PR-5%, Stable disease-52%	[145]
NCT01604772	II	Recurrent/stage IV ADCC	MK2206	None	Completed	CR/PR–0% Stable disease-81%	[146]
NCT05172245	I	Stage III-IVB HNSCC	Ipatasertib	Cisplatin /Radiation	Not yet recruiting	–	[147]

## Appendix A

### Appendix A.1. Cell Lines

HaCaT: Normal skin keratinocyte (Squamous epithelial origin). The first reason for selecting the HaCaT cell line is the unavailability of oral mucosal keratinocytes in the laboratory. Although derived from skin squamous epithelial region their morphology and function are similar to oral mucosal squamous epithelial cells [163].

### Appendix A.2. TYS

Oral adenoid squamous cell carcinoma (an epithelial neoplastic cell line was derived from well-differentiated squamous cell carcinoma that arose in human oral mucosa). The cell line was then treated with sodium-butyrate and transplanted into nude mice. The resulting mass was then histopathologically tested and interpreted as a human adenoid squamous cell carcinoma derived from the minor salivary gland present in oral mucosa.

### Appendix A.3. Wound Healing Assay

As tumours have been described as wounds that never heal [164], we conducted a wound healing assay (directed 2D migration assay) for investigating both single and collective cell migration. Cells were cultured until a monolayer was developed. The cell monolayer was then serum-starved overnight, and a gap was made manually in the monolayer using a 100 µL pipette tip. The cells were then incubated in 10 ng/mL (optimised) of different growth factors for 24 h. Images were captured randomly at t = 0 and t = 24 h by an inverted microscope (Olympus IX70, Osaka, Japan) at 200× magnification to monitor the cell migration causing gap closure. Images were processed by Olympus CellSense software. Cells treated with serum-free (SF) medium were used as negative control.

### Appendix A.4. Scatter Assay

Cell scattering is a dynamic process for investigating single cell migration via epithelial to mesenchymal transition (EMT) in which dispersion of compact colonies of epithelial cells is induced by certain soluble factors such as growth factors [165]. A total 4 × 104 cells were seeded per 60 mm dish and grown until well-defined colonies (maximum 20 cells in a colony) were visible. The cells were then washed twice, serum starved overnight, and incubated with the stated growth factors (10 ng/mL) diluted in SF medium for 24 h. Scattering of the cells was observed by at least two individuals before images were taken randomly. Images were captured using an Olympus inverted microscope at 200× magnification and processed by CellSense software.
